# The Rapunzel syndrome: a hairy tale

**DOI:** 10.1186/s40792-023-01631-w

**Published:** 2023-03-28

**Authors:** Luca Schuler, Martina Hodel, Claudia Stieger

**Affiliations:** 1 Department of Emergency Medicine, Cantonal Hospital of Lucerne, Lucerne, Switzerland; 2 Psychiatric Clinic of Lucerne, Lucerne, Switzerland; 3 Surgical Department, Cantonal Hospital of Lucerne, Lucerne, Switzerland

**Keywords:** Trichobezoar, Bowel obstruction, Trichophagia, Obsessive–compulsive disorder, Rapunzel syndrome

## Abstract

**Background:**

Trichobezoars are a rare medical condition, often requiring a surgical approach and commonly associated with an underlying psychiatric disorder. The Rapunzel syndrome is a rare variant of trichobezoar in the stomach extending from the stomach into the small intestine causing a bowel obstruction.

**Case presentation:**

In this case report, the clinical presentation, diagnostic approach, and surgical removal of a large-size bezoar (Rapunzel syndrome) in a young and otherwise healthy female is described. Different surgical strategies are discussed. Psychiatric exploration gives an insight on development of trichophagia ultimately leading to the forming of the trichobezoar.

**Conclusions:**

This brief report sheds light on the importance of the collective mind of a multidisciplinary team preventing a potentially fatal outcome.

## Background

A bezoar is a concrement of indigestible human components or vegetable fibers that accumulate over time in the gastrointestinal tract. The most common type of bezoar in humans is the trichobezoar, which is mostly made of hair. However, bezoars can also form from any indigestible material. Various case reports describe the occurrence and diagnostic as well as surgical management of these peculiar surprises.

Trichobezoars, on the contrary to other bezoars, are not associated with alterations in the gastrointestinal motility, but with underlying psychiatric disorders. They are most commonly presented in young female adults [1]. The development of trichobezoars is a salient complication of trichophagia, an obsessive–compulsive behavior characterized by eating hair [2]. Trichophagia is thought to be in most cases preceded by trichotillomania, an irresistible urge to pull one’s own hair [3]. However, other underlying or associated psychiatric diseases involve post-traumatic stress disorder (PTSD), for example as a result of childhood neglect or abuse, as well as affective disorders [4, 5]. While diagnostic and surgical procedures of trichobezoars are well described in the literature, psychiatric literature on the etiology of trichobezoars remains anecdotal and unsystematic [6].

The here reported rare and unusual form of a trichobezoar extending into the small intestine is colloquially called “Rapunzel syndrome”.

## Case presentation

We are reporting about a previously healthy 21-year-old female with an ileus due to a large-size bezoar in the stomach and small bowel after a history of eating her own hair for several years.

**Fig. 1:**
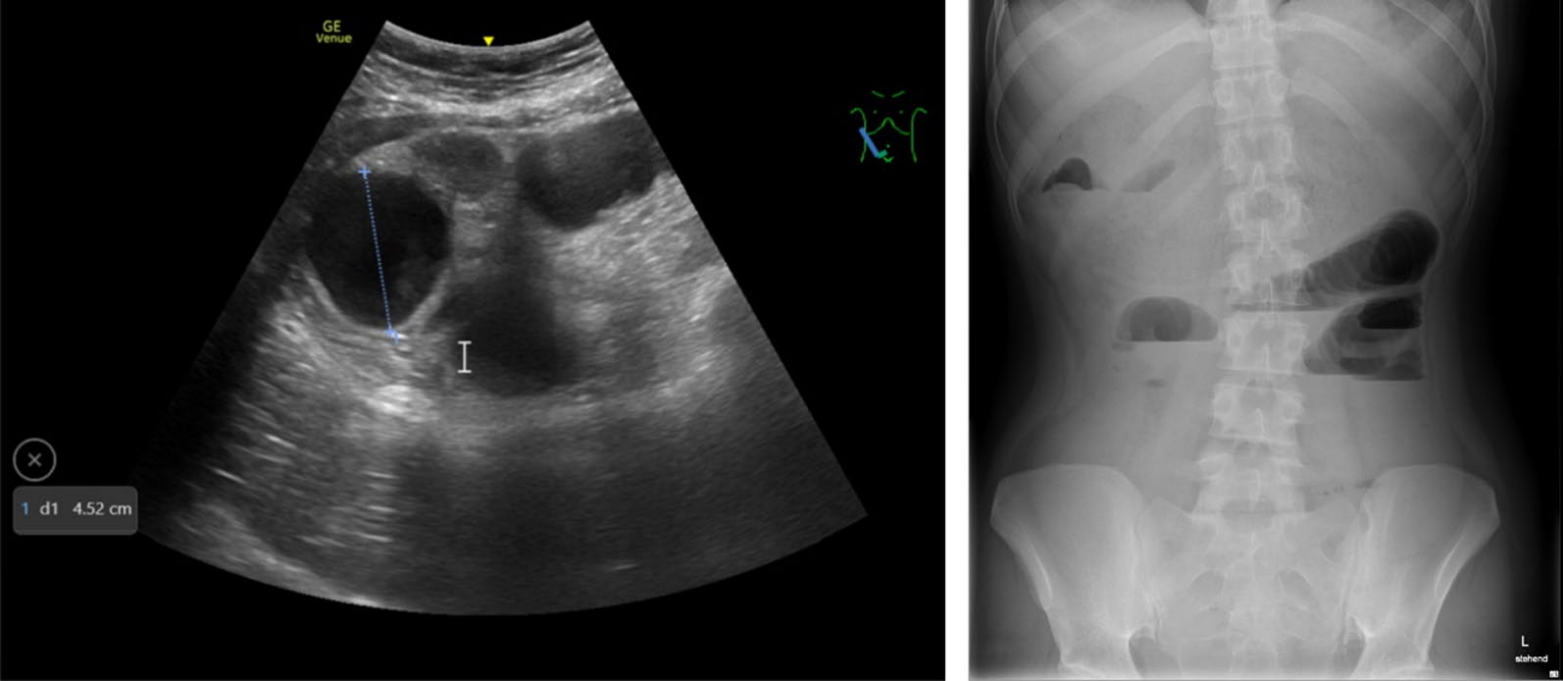
Ultrasound of the lower abdomen shows enlarged small bowel (4.5 cm diameter) consistent with an ileus. An obstructing conglomerate tumor is visible (left side). Plain radiograph shows multiple air fluid levels as a sign for ileus (right side)

The woman presented to the emergency department with a history of unspecific abdominal pain and vomiting after food or water intake. Furthermore, similar but less severe symptoms were reported since a few years. According to the mother, a habit of eating hair was observed by family members. A mild anemia was previously supplemented by intravenous iron treatment. Diagnostic GIT-endoscopy was pending. Apart from that, medical history was unremarkable, and the patient never had abdominal surgery before. The patient presented with normal weight and shoulder long hair. Abdominal exam showed reduced bowel sounds with otherwise normal findings. No abdominal tenderness was noted. Ultrasound showed pendulum peristalsis in the small bowel, a greatly enlarged stomach and a non-vascularized obstructing mass in the lower abdomen (Fig. 1). Plain radiograph of the abdomen showed multiple air fluid levels with distended small intestinal bowel loops (Fig. 1). Laboratory work-up revealed a leukocytosis (19.8 G/L, ref 2.6–7.8) and a hyperregenerative microcytic and hypochromic anemia (95 g/L, ref 115–148). C-reactive protein (CRP)-levels, complete metabolic panel, as well as liver and pancreatic enzymes, were normal. A urine sample was contaminated and therefore non-conclusive.

**Fig. 2:**
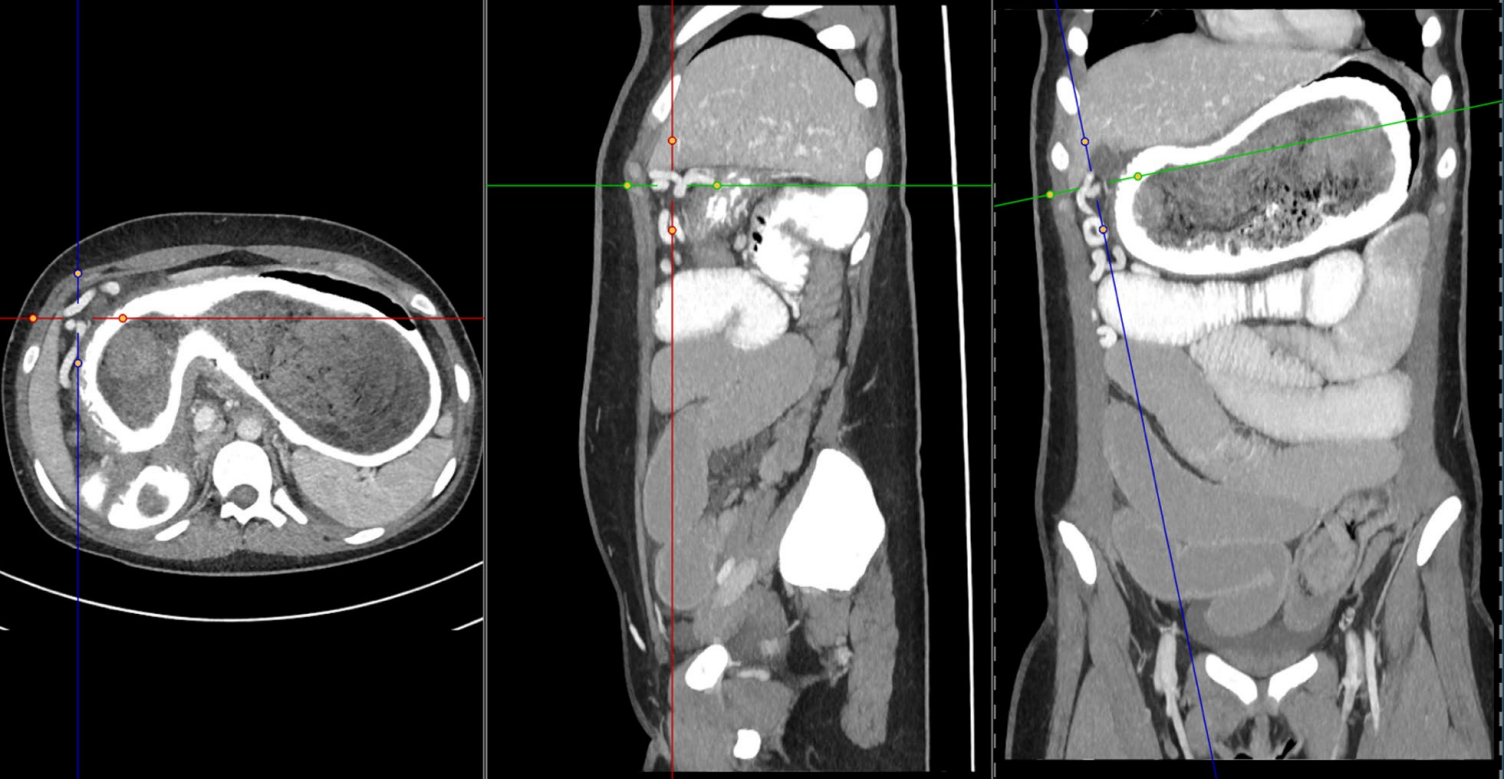
CT scan of the abdomen shows a big bezoar in the stomach and a smaller one in the small intestine. The crosshairs points at the extensive venous collateral circulation

**Fig. 3:**
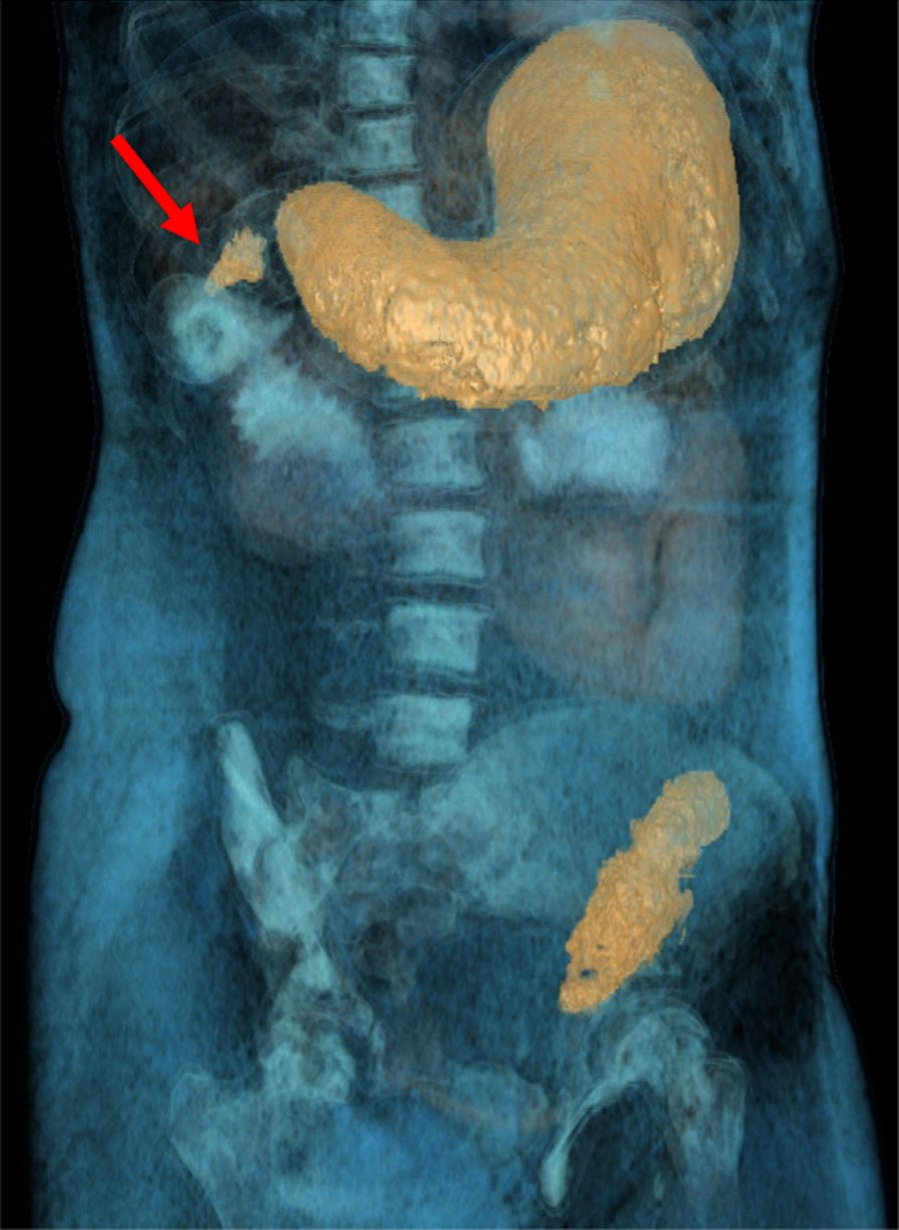
Virtual CT-reconstruction of the bezoar in the stomach and the small intestine. The marked area (red arrow) indicates a possible extension into the small intestine

Because of young age of the patient, computed tomography (CT) scan was discussed considering exposure to radiation versus direct explorative laparoscopic surgery with a high risk for laparotomy. It was decided to perform a CT scan nevertheless due to the ileus-like picture of the abdomen and the repetitive vomiting to evaluate extent of the likely needed operation. CT scan confirmed the suspected diagnosis of a mechanical-caused ileus due to a large mass in the small intestines in the left lower abdomen. Furthermore, there was a large mass seen distending the whole stomach (Figs. 2, 3).

Conclusively, a mechanical ileus due to a bezoar in the small intestine and a bezoar in the stomach was diagnosed. Additionally, extensive collateral circulation with portacaval shunting was present, most likely due to compression of the portal vein (Fig. 2).

**Fig. 4:**
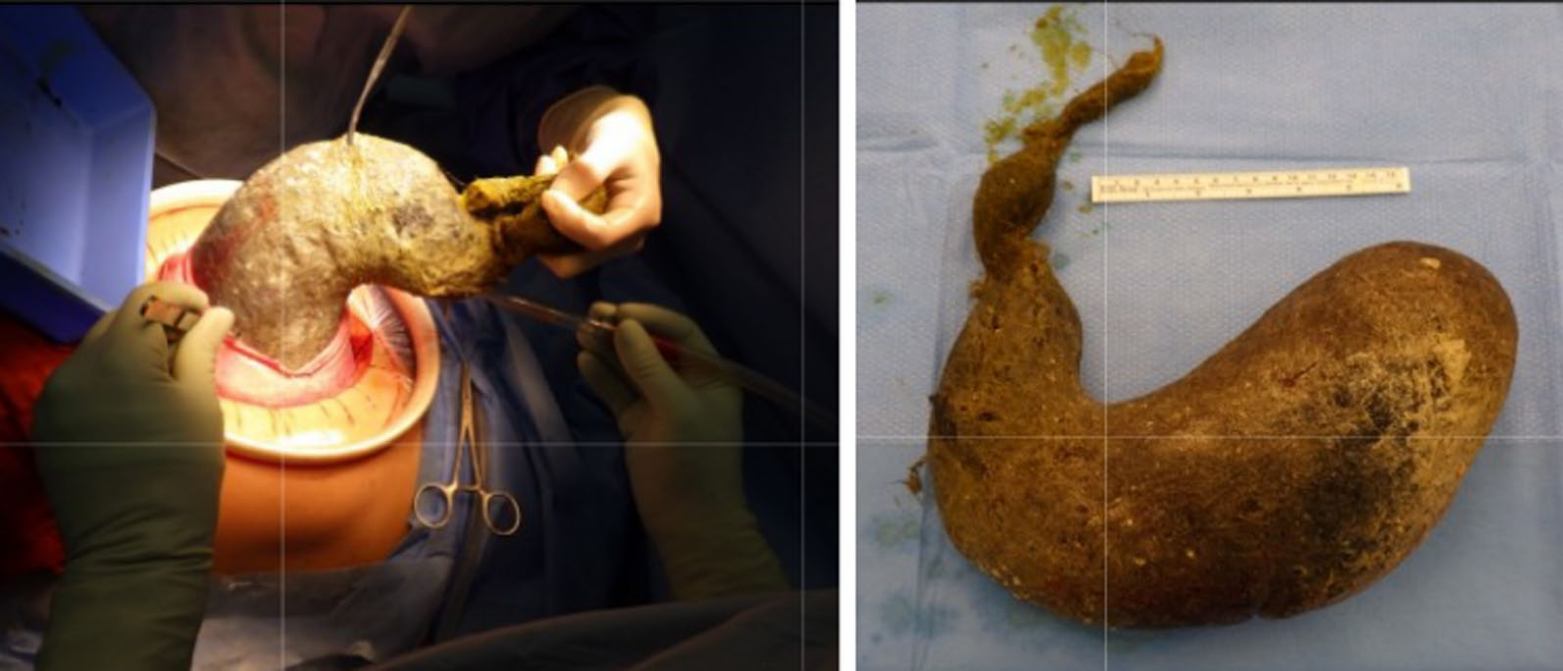
A stomach-shaped trichobezoar with a tapering tail extending into the small bowel was surgically removed en bloc by an upper median laparotomy and gastrotomy (added ruler equals 15 cm)

A virtual CT-reconstruction of the findings initially showed a possible tapering tail reaching from the stomach downwards (Fig. 3). This finding was consistent with subsequent intraoperative findings (Fig. 4).

**Fig. 5:**
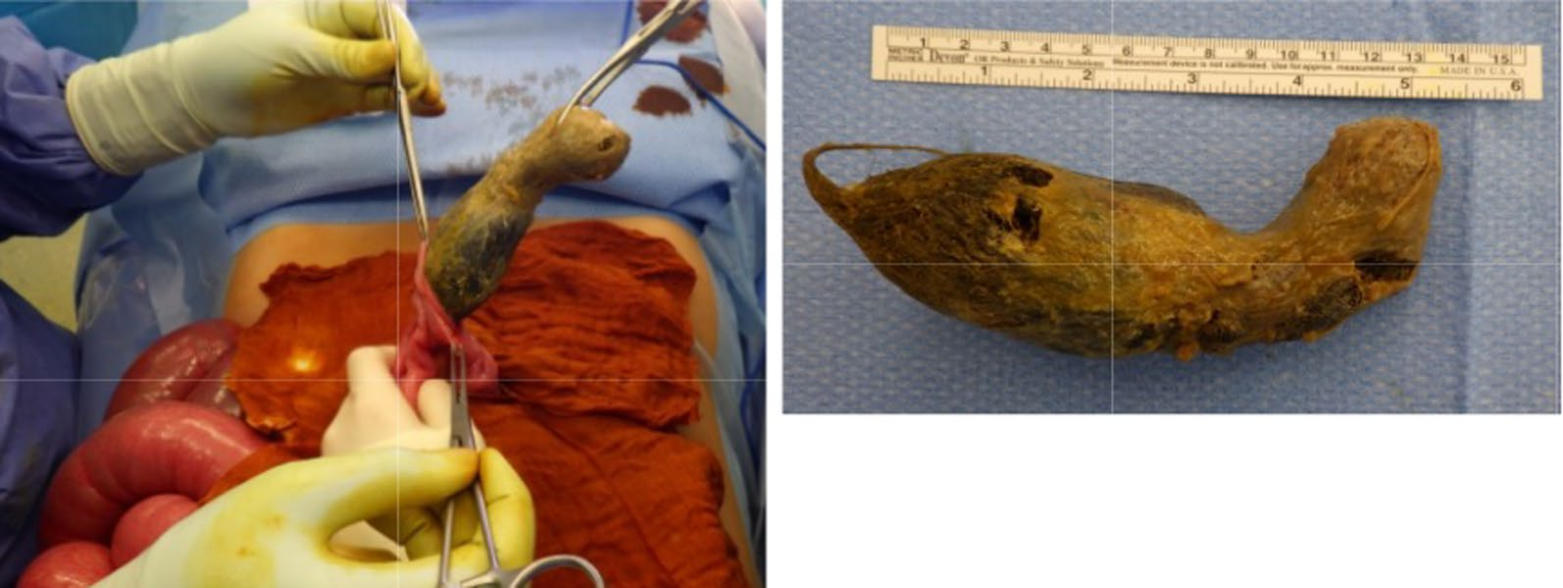
A smaller trichobezoar with a tail was surgically removed en bloc by an upper median laparotomy and ileotomy (added ruler equals 15 cm)

A primary upper median laparotomy with gastrotomy and ileotomy was performed and a 29*19*10 cm trichobezoar was removed from the stomach (Fig. 4) and a smaller 14*4*4 cm trichobezoar was removed from the small intestine (Fig. 5), each in one piece. The trichobezoar removed from the stomach showed a tapering tail extending into the small bowel and was a perfect cast of the stomach, pylorus and duodenal bulb (Fig. 4).

Trichophagia was diagnosed by the in-house psychiatric staff. In psychiatric exploration, the patient reported having memories of "playing with her own hair" since the age of five after observing her mother showing similar behavior. The patient reported increase in pulling hair with subjective stress level. While the patient initially stored the pulled out hair in the nightstand, swallowing started after family members became aware of it. While the patient described herself as socially rather isolated and with only a few friends, there were no obvious signs of psychopathology. Especially denied were mood disorders, anxiety or/and a history of abuse or neglect, commonly reported in patients with trichophagia [7]. However, the patient reported suffering from severe sleep disorders, which she attributed to nausea and stomach cramps during the night.

The patient further described hair pulling and swallowing possessing stress relieving qualities, albeit being performed mostly secretly at home and not in public. Previous attempts of stopping the compulsive acts have failed. The patient furthermore described hair pulling and swallowing as happening somehow “out of her own conscious perception”. Even though the patient realized that there was something “out of order” with appetite and digestion, she did not consciously attribute the symptoms to trichophagia.

The patient was discharged 5 days after admission after a clinically good recovery but against surgeon’s recommendation due to still highly elevated CRP-levels (202 mg/L, ref < 5). Due to the extent of trichophagia and lack of insight into severity of the disease, a specialized in-patient psychiatric clinic to treat the obsessive–compulsive disorder was recommended but rejected by the patient. An out-patient appointment was organized. In a follow-up psychiatric evaluation, the patient stated that she has continued eating hair after hospital discharge. The patient perceived eating hair as “hard to control” and “happening rather unconsciously", followed by frustration and a feeling of failure.

A check-up with the family doctor showed good wound healing, decreasing CRP-level and increasing hemoglobin levels.

## Discussion

Foreign material in the gastrointestinal tract can lead to Bezoars. These concrements occur mainly in the stomach. Bezoars composed of hair or hair-like fibers are called trichobezoars. They are associated with the obsessive–compulsive disorder trichotillomania (pulling out one’s own hair) and trichophagia (eating hair), however, there is anecdotal evidence of other (comorbid) underlying psychiatric diseases such as affective disorders as well as severe neglect or (sexual) abuse [7, 8]. According to estimations, only 1% of patients with trichophagia develop a trichobezoar [9, 10]. Trichobezoars form when hair in the stomach escapes the peristaltic propulsion of the stomach due to its slippery surface and accumulates in the folds of the gastric mucosa. In rare cases, a higher amount accumulates. As a result, the gastric peristaltic is forming the mass into a ball and ultimately into a perfect cast of the stomach, usually as one single solid mass [11].

In literature, it is assumed that the usual location of these trichobezoars is associated to the holdup by the pylorus, the motion of the stomach and, finally, the entangling of new hair into the mass. Stomach mucus covers the trichobezoar and gives it a shiny look; gastric acid denatures the hairs’ protein and gives the bezoar its dark color. Due to decompensation and fermentation of the hair, the patients might have a putrid breath and sometimes present with halitosis [12, 13].

Rapunzel syndrome is a rare form of a trichobezoar with no consistent definition in literature. Various definitions are described, for example a gastric trichobezoar with a tail extending to the ileocecal junction [14, 15]. Furthermore, some authors describe it as a simple gastric trichobezoar with a tail which may lead to the jejunum or further and some define it as any size which causes intestinal obstruction [14].

Patients usually stay asymptomatic for many years. Symptoms start developing as the trichobezoar increases in size up to the point of obstruction. The most common symptoms are therefore abdominal pain, nausea and vomiting, intestinal obstruction, and peritonitis. Indirect signs, caused by malabsorption, are iron deficiency with consecutive microcytic and hypochromic anemia, vitamin B12 deficiency with consecutive megaloblastic anemia, fatigue, protein-losing enteropathy, and weight loss. A large obstructing or eroding bezoar may cause complications such as gastric ulceration, obstructive jaundice and acute pancreatitis [16].

When a bezoar is suspected, the focus in examination should be on trichotillomania and trichophagia as well as ingestion of items such as dolls/wigs or pet hair. Further clues are a refractory halitosis and patchy alopecia. The gold standard for diagnosis is upper gastrointestinal endoscopy for visualizing as well as possible sample taking and, when confirmed, initiation of therapeutic options.

The treatment of a bezoar focuses on surgical removal of the mass. Prevention of recurrence may only be reached by addressing the underlying psychiatric illness. The removal of the mass depends on its consistency, size and localization: the right approach might be via endoscopy or surgery. The endoscopic approach might be effective for phytobezoars or lactobezoars as they are usually smaller in size. Specialized bezotomes and bezotriptors (medical device to pulverize bezoars either mechanically or acoustically) are used to fragment solid trichobezoars [17]. Trichobezoars, particularly large ones (> 20 cm), and Rapunzel syndrome are less likely to be removed via endoscopy and usually require surgery due to their extension [9]. Surgical removal is done by gastrostomy and enterotomy. Surgery is indicated due to the size of the bezoar causing perforation, hemorrhage, or an ileus. The surgical access depends on the trichobezoars’ size by performing an upper midline laparotomy with gastrotomy, as performed in our patient, or a laparoscopic approach in minimal invasive approach for smaller to moderate-size bezoars [14]. Multiple other methods like extracorporal shock wave lithotripsy, administration of enzymes to the stomach (pancreatic lipase, cellulose), and medications (metoclopramide, acetylcysteine) demonstrate heterogeneous treatment success [17, 18].

Recurrence is reported after the initial removal of bezoars. Therefore a long-term psychiatric follow-up is advised [19]. However, the patient’s motivation to engage in psychiatric/psychological treatment (e.g., cognitive–behavioral therapy to reduce obsessive compulsive behavior) is a prerequisite.and essentially preventing recurrence of a trichobezoar. In that case, the long-term prognosis in these cases is favorable [16].

## Conclusions

The Rapunzel syndrome as presented in this case is a rare variant of trichobezoar in the stomach extending from the stomach into the small intestine and/or causing a bowel obstruction. While small trichobezoars may be removed by an endoscopic approach (fragmentation, lavage, enzymatic therapy, or combinations), larger trichobezoars/the Rapunzel syndrome usually needs a surgical removal.

Trichobezoar as an entity should be considered in the differential diagnosis of abdominal pain and non-tender abdominal mass in young patients. A thorough assessment of psychiatric history is mandatory to address the underlying disease to prevent recurrence.

## Data Availability

Not applicable.

## Abbreviations

PTSD: Post-traumatic stress disorder
GIT: Gastrointestinal tract
CRP: C-reactive protein
CT: Computed tomography
